# Facet-Dependent Restructuring and Catalytic Activity of Cu Single Crystals during CO Electro-Oxidation

**DOI:** 10.1021/acsnano.5c13881

**Published:** 2025-12-09

**Authors:** Matthias Leitner, Francesc Valls Mascaró, Andrea Auer, Julia Kunze-Liebhäuser

**Affiliations:** Institute of Physical Chemistry, 27255 University Innsbruck, Innrain 52c, Innsbruck 6020, Austria

**Keywords:** electrochemical scanning tunneling microscopy, CO electro-oxidation, structure−activity relationship, surface restructuring, Cu single crystals

## Abstract

Understanding the relationship between the surface structure and catalytic activity is central to the rational design of efficient electrocatalysts. While Cu is well-known for its tendency to restructure under reaction conditions, it remains poorly understood how exactly such dynamic structural changes influence the catalytic activity. Here, we combine electrochemistry with *in situ* electrochemical scanning tunneling microscopy (EC-STM) to study and directly compare CO electro-oxidation on Cu(111) and Cu(100) single crystals and how it relates to the structural changes on these two faces. We find that both surfaces undergo nanometer-scale restructuring during the reaction, leading to the formation of undercoordinated Cu adatoms, which act as the catalytically active sites for both surfaces. However, their morphological evolution differs markedly: Cu(111) exhibits dynamic and reversible restructuring, maintaining a high density of adatom nanoclusters across successive potential steps, whereas Cu(100) forms clusters that evolve less reversibly with a gradual decrease in cluster density over time and upon repeated potential steps. Notably, the evolution of clusters and their density do not directly correlate with the observed catalytic activity for either facet. Instead, we propose that the facet-dependent differences in activity stem primarily from variations in the effective density of the catalytically active Cu adatoms and their distinct interaction with reactants rather than from different structural motifs. These findings highlight the crucial role of dynamic surface restructuring in governing catalytic performance and emphasize the need to account for facet-specific morphological and structural changes in the rational design of efficient Cu-based electrocatalysts.

## Graphical abstract

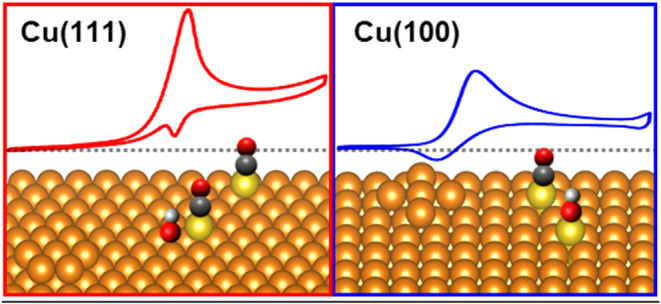

Owing to its role as a key reaction intermediate, carbon monoxide (CO) and its interaction with atomically well-defined metal surfaces, as well as its electro-oxidation, particularly on Pt^1–4^ and Au^5–10^ single crystals, have been extensively investigated. These studies are fundamental to the mechanistic understanding of more complex electrocatalytic oxidation reactions of high-energy-density fuels, such as alcohols.^11^ A detailed understanding of CO electro-oxidation is therefore essential for the rational design of advanced electrocatalysts for energy conversion and storage applications. More recently, it has been demonstrated that earth-abundant Cu can also efficiently oxidize CO in alkaline media,^12–14^ which is accompanied by strong and continuous surface structural changes under reaction conditions.^12^

Surface restructuring and dynamic morphological changes are well-known characteristics of Cu electrodes and are mainly related to their relatively low cohesive energy.^15–23^ We have previously shown that Cu(111) electrodes begin to reconstruct in alkaline media already at the potential of zero free charge.^24^ In acidic electrolytes under cathodic conditions, hydrogen-induced surface reconstructions have been reported for both Cu(111)^25^ and Cu(100),^26^ while a CO-promoted restructuring was reported under CO^27^ and CO_2_ ^16,18–22,28^ electroreduction conditions in both neutral and alkaline media using electrochemical scanning tunneling microscopy (EC-STM).

In a CO-containing alkaline electrolyte at anodic potentials, *i.e*., where CO electro-oxidation occurs, Cu(111) undergoes largely reversible nanoscale morphological changes, resulting in the formation of small Cu adatom clusters. A combination of EC-STM with first-principles microkinetic modeling has demonstrated that these low-coordinated Cu surface species play a crucial role in the CO electro-oxidation process.^12^ These surface restructuring phenomena and the interplay between cluster formation and self-activation are well-known for Cu in both electro- and gas-phase catalysis. A prominent example of this type of reconstruction at the solid–gas interface is observed during the water–gas-shift reaction. The surface morphology evolution of Cu single crystals and their activation toward this reaction have been studied at different elevated CO pressures (up to ∼130 mbar) using high-pressure STM and ambient pressure X-ray photoelectron spectroscopy.^15,23,29^ On Cu(111), triangular and hexagonal nanoclusters with diameters of approximately 0.5 nm (3 Cu atoms) and 1.5 nm (19 Cu atoms), respectively, are formed, whereas for Cu(100), square-shaped nanoclusters consisting of five Cu atoms appear at CO pressures as low as ∼0.25 mbar. At higher CO pressures, the Cu(111) surface is completely covered with clusters that are closer to each other and larger in size,^15^ whereas on Cu(100), the squared nanoclusters evolve into one-dimensional structures, three atoms wide and aligned along the [001] crystallographic direction.^23^ These studies clearly highlight the pronounced facet-dependent restructuring characteristics of low-index Cu surfaces under varied elevated CO pressures, which is highly relevant for heterogeneous catalysis, as exemplified by an enhanced activity of the clustered surfaces toward the water–gas-shift reaction.

While facet-dependent catalytic activity is also widely recognized in electrocatalysis, structure–activity relationships are often constructed under the implicit assumption of static, idealized surfaces. However, it is increasingly evident that catalytic surfaces dynamically restructure under reaction conditions and that these restructuring processes are themselves strongly facet-dependent. This introduces a critical yet often overlooked dimension to the understanding of structure–activity correlations.

Here, we present a direct comparison of Cu(111) and Cu(100) in terms of their structural and morphological evolution during CO electro-oxidation, using EC-STM, to explicitly elucidate the interplay between facet-dependent restructuring behavior and catalytic activity. We show that surface restructuring occurs on both Cu(111) and Cu(100) under CO electro-oxidation conditions. Together with complementary electrochemical measurements, our results demonstrate that while both facets exhibit extensive nanoclustering, the size and density evolution of those clusters with time and applied potential do not directly correlate with the observed catalytic activity. Therefore, we conclude that the active species on both facets are highly mobile and extremely small Cu adatoms and not the clusters that are formed and readily observable in EC-STM. Accordingly, the observed facet-dependent catalytic performance arises primarily from differences in the effective density of these small, presumably single-adatom active Cu sites and their specific interactions with reactants rather than from different structural motifs.

This facet-dependent reconstruction behavior underscores the complex interplay between surface morphology and structure and electrocatalytic activity, challenging simplified structure–activity models that assume static surface structures.

## Results and Discussion

To assess the overall electrocatalytic performance of Cu(111) and Cu(100) toward CO electro-oxidation, cyclic voltammetry was performed, and the results are presented in Figure 1. In the CO-saturated electrolyte, both low-index Cu single-crystal facets exhibit a distinct anodic peak with high current density, which is absent under CO-free conditions and results from CO electro-oxidation. This is consistent with previous reports.^12–14^ A comparison of the overpotentials (η), defined as the difference between the equilibrium potential (*E* ^0^)­for CO oxidation (−0.1 *V*_RHE_) and the potential at which a current density of 0.1 mA cm^–2^ is reached, reveals a lower η for Cu(100) (100 mV) than for Cu(111) (180 mV). While Cu(100) exhibits a lower overpotential, its anodic current densities during CO oxidation, determined within the quasi-steady-state region at 0.3 *V*_RHE_, are also significantly lower (0.07 versus 0.12 mA cm^–2^). Interestingly, for Cu(111), the CO electro-oxidation exactly coincides with the OH^–^ adsorption that is typically observed in CO-free NaOH electrolyte on both facets and that is characterized by a sharp peak on Cu(111) (Figure 1a),^24,30–33^ whereas Cu(100) (Figure. [Fig fig1]b) shows a clear potential shift of approximately 0.15 V between the OH^–^ adsorption peak and the CO oxidation peak. A closer inspection of the current responses with and without CO suggests that the anodic peak in the CO-containing NaOH arises from a convolution of OH^–^ adsorption and CO oxidation processes, as evidenced by the similarities of the small, and in the case of Cu(111), sharp, desorption peaks in the cathodic sweeps. Interestingly, this coincidence of the anodic peaks is observed for both Cu(111) and Cu(100), even though the respective OH^–^ adsorption processes have different initial potentials on the two faces (see Figure 1). Since the peak responses include contributions of multiple processes, further evaluation of the electrocatalytic activities and the reversibility of the processes is conducted via current transient measurements, which are shown in Figure 2. For the potential step that initiates each transient, we selected the peak potential (*E*_p_) at +0.1 V to ensure comparability. These current transients depict clear differences between Cu(111) and Cu(100). As shown in Figure 2a, both facets exhibit stable and reversible steady-state current densities over multiple potential steps into the CO electro-oxidation regime (7–8) steps over 35 to 60 min. Cu(111) consistently shows approximately twice the current density compared with Cu(100), and the steady-state is reached more rapidly after each potential step (Figure 2b).

**Figure 1:**
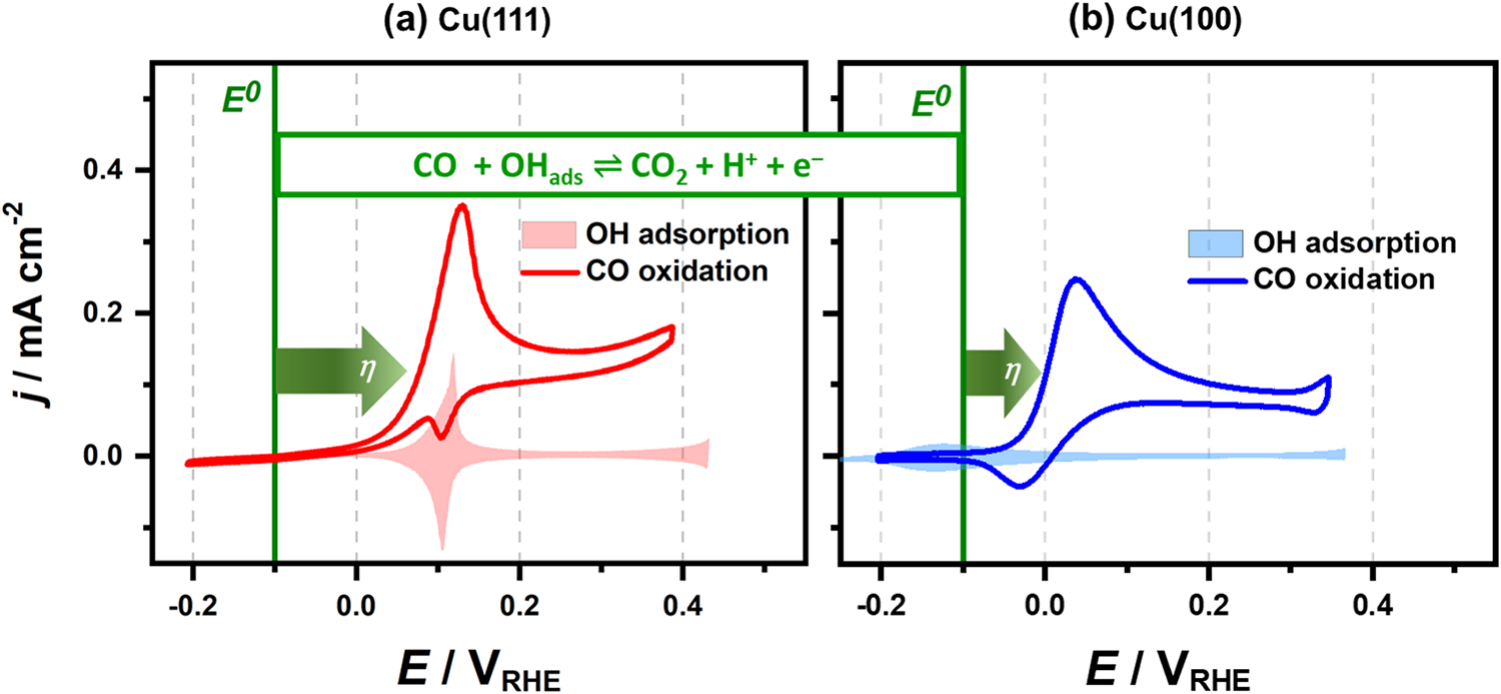
CO electro-oxidation on Cu­(hkl) single crystals. Cyclic voltammograms (CVs) of (a) Cu(111) (red) and (b) Cu(100) (blue) recorded in 0.1 M NaOH (pH 13), with (solid lines) and without (shaded areas) CO in the electrolyte. Scan rate: 50 mV s^–1^. Current density (*j*) is normalized to the geometric surface area. Potentials are referenced to the reversible hydrogen electrode (RHE).

**Figure 2:**
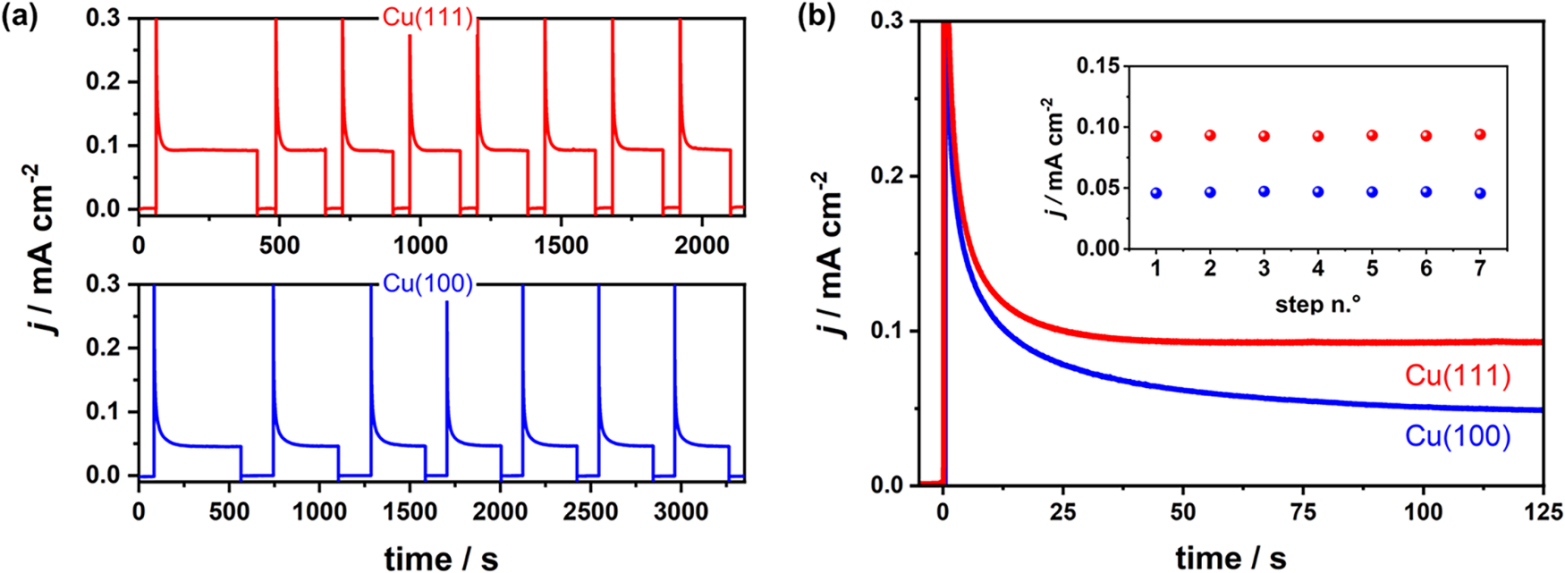
Current transients of Cu(111) (red) and Cu(100) (blue) during repeated potential steps into the CO electro-oxidation regime. (a) Multiple consecutive current transients to 0.2 V (Cu(111)) and 0.12 V (Cu(100)) were used to evaluate the catalytic activity of each facet. (b) Overlay of a representative transient for each surface to compare the evolution of the current response. The inset shows steady-state current densities as a function of the step number.

The transients in Figure 2 reveal clear differences between Cu(111) and Cu(100) in both steady-state current densities and current decay characteristics. Initially, we attributed these differences to normalization by geometric area alone, which overlooks the different surface atom densities of the (111) and (100) facets and therefore does not provide a meaningful comparison of intrinsic activity. To address this, we additionally normalized the current densities by the surface atom densities of the unreconstructed (1 × 1) surfaces (Figure S1 in the Supporting Information). This analysis confirms that even after accounting for the lower surface atom density of Cu(100), it still exhibits a significantly lower current density per surface atom. This difference in per-atom activity is consistent with previous reports of substantial surface restructuring during CO electro-oxidation on Cu(111),^12^ where the formation of Cu adatoms and small clusters creates undercoordinated active sites, effectively increasing the number of catalytically relevant atoms beyond the intrinsic surface atom density. To better understand the reasons for these differences in intrinsic activity, EC-STM studies were conducted on both Cu(111) and Cu(100) to directly image and correlate structural and morphological changes during CO electro-oxidation.

STM images of the Cu(111) and Cu(100) surfaces at −0.2 *V*_RHE_, where no CO electro-oxidation occurs, reveal a morphology characterized by atomically flat, micrometer-scale terraces separated by monatomic steps that are characteristic of metallic Cu (Figure 3a,e). For the first potential steps, *E*_p_ + 0.1 V was chosen for both surfaces to ensure comparability with the transients. Upon stepping into the CO electro-oxidation regime, pronounced changes in the surface structure and morphology are observed.

**Figure 3:**
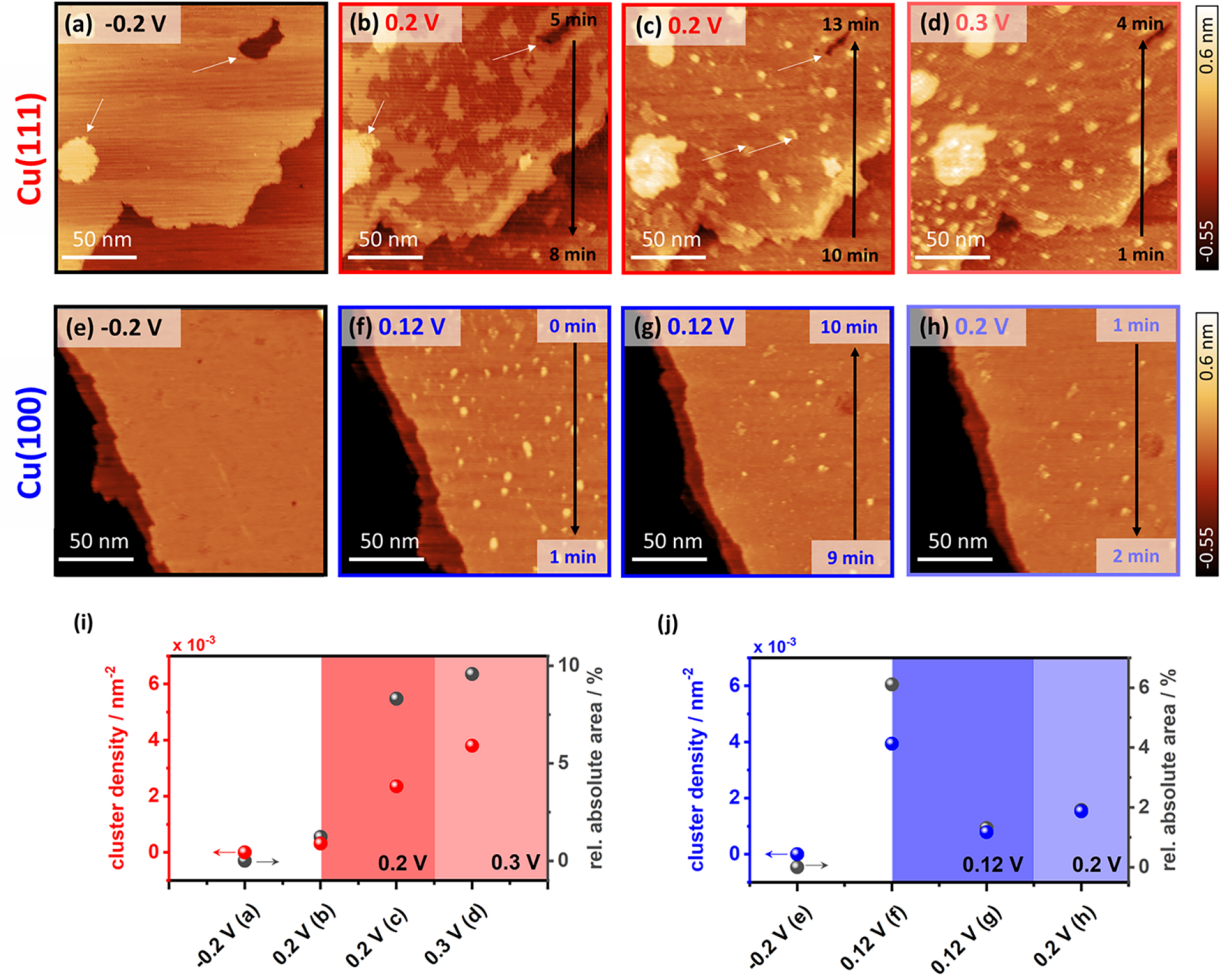
Time- and potential-dependent imaging of Cu(111) and Cu(100) during CO electro-oxidation. EC-STM images of Cu(111) in CO-saturated 0.1 M NaOH at (a) −0.2 *V*_RHE_ and (b–d) during a potential step to 0.2 *V*_RHE_, where CO oxidation, OH^–^ adsorption, and surface reconstruction occur. Corresponding images of Cu(100) are shown at (e) −0.2 *V*_RHE_ and (f–h) during the potential step to 0.12 *V*_RHE_. White arrows indicate the growth of an adatom island and the shrinkage of a vacancy island (a, b), as well as the formation of Cu adatom clusters (c, d, f). All image sizes are 180 × 180 nm^2^. *I*_tip_ = 1 nA (a–d), 1.55 nA (e, f), 1.06 nA (g, h); *E*_tip_ = 0.15 V. Quantitative cluster analysis of the shown EC-STM images for (i) Cu(111) and (j) Cu(100), displaying cluster density (left *y*-axis) and relative cluster area (right *y*-axis). Colored arrows indicate the corresponding *y*-axis.

On Cu(111), dark patches appear in the middle of the terrace immediately after the potential step (Figure 3b). This is accompanied by terrace expansion, as evidenced by the growth of an adatom island and the shrinkage of a vacancy island (white arrows in Figure 3a,b). The dark patches exhibit a depth of around 90–100 pm, which corresponds to approximately half a monatomic step height (see Figure S2). They are attributed to the adsorption of hydroxide (OH^–^) ions, which locally reduce the conductance, consistent with previous reports.^34,35^ This is further supported by the reference measurement in CO-free NaOH (Figure S3), which also shows these patches appearing at 0.2 *V*_RHE_. There, they are smaller and more uniformly distributed, suggesting that in the CO-containing electrolyte, CO coadsorbs and preferentially blocks parts of the surface close to the step edges. Thus, OH^–^ adsorption is restricted to the center of the terrace, which is consistent with similar observations in the presence of chlorides.^36^ Additionally, in the absence of CO, OH^–^ adsorption appears to be kinetically hindered, as indicated by the delayed appearance of dark spots (Figure S3c), while in the presence of CO, they form more readily (Figure 3b). This suggests enhanced kinetics or reinforced (lateral) interactions, as was proposed for Au(111).^8,10^ With time, Cu adatom nanostructures and small, partially threadlike clusters become visible within the dark areas. As the dark patches expand, these clusters increase in density and, to some extent, in size with a final height of one monolayer of Cu (Figure 3c). Quantitative cluster analysis of the EC-STM images, shown in Figure 3i, reveals a gradual increase in cluster density with an average cluster size of 3–5 nm, reaching a density of 0.004 nm^–2^ upon increasing the potential to 0.3 *V*_RHE_ (Figure 3d). The relative absolute area, representing the fraction of the surface covered by clusters, follows a similar increasing trend and reaches values of 9.6% at 0.3 *V*_RHE_. Consistent with previous results,^12^ we attribute the observed nanoprotrusions to CO-stabilized Cu adatom clusters, which are absent in Ar-saturated NaOH (Figure S3). At this lower overpotential, we capture the early stages of OH-induced and CO-promoted surface restructuring, which were not resolved earlier.^12^ Due to the expansion of the first Cu layer and the resulting lower surface atom density, excess Cu atoms are ejected and stabilized on the surface by CO. Without CO, these adatoms contribute to terrace growth via step attachment, and the dark OH^–^ adsorption patches still visibly cover the terrace (Figure S3d).

On Cu(100), Cu adatom clusters with a monatomic height (see Figure S4) appear immediately after the potential step and are homogeneously distributed across the terraces (Figure 3f). Quantitative cluster analysis (Figure 3j) shows an initial cluster density of 0.005 nm^–2^ with an average size of 3–4 nm, directly after the step to 0.12 *V*_RHE_. Similar to Cu(111), the average cluster size remained largely unchanged. However, in contrast to Cu(111), the cluster density on Cu(100) decreases with time. Upon increasing the potential to 0.2 *V*_RHE_ (Figure 3h), the cluster density initially shows a slight increase before decreasing again (data not shown). Interestingly, stepping into the OH^–^ adsorption regime in Ar-saturated 0.1 M NaOH also induces the formation of small adatom clusters on Cu(100); however, these exhibit smaller sizes and slightly higher densities compared to the clusters formed during CO electro-oxidation (Figure S5). These findings suggest that while on Cu(111), the stabilization of high-energy nanoclusters requires CO adsorption at undercoordinated sites, on Cu(100), similar features are already stabilized by OH^–^ adsorption alone, with CO leading to slightly larger clusters. Notably, only in the CO-free electrolyte do a few small dark patches appear on Cu(100) (90–100 pm in height, see Figure S5), which grow over time and are attributed to OH^–^ adsorption, as explained above for Cu(111).

These observations provide valuable insights into the structure–activity relationships of low-index Cu surfaces during CO electro-oxidation. On Cu(111), the nanocluster density gradually increases with time and applied electrode potential (Figure 3i), whereas on Cu(100), the initial cluster density generally decreases under similar conditions (Figure 3j). Despite these contrasting structural evolutions, the current transients for both surfaces, shown in Figure 2, stabilize at distinct steady-state current densities after approximately 3 min. This behavior contrasts with the ongoing morphological changes observed within the same time frame, where no stable cluster density is reached, even after at least 10 min of imaging for either facet. Steady-state current densities also remain constant at elevated potentials, as previously shown for Cu(111) at 0.3 *V*_RHE_ (ref 12). Interestingly, Cu(111) exhibits steady-state current densities higher than those of Cu(100), even after normalization to the (1 × 1) surface atom density, despite the higher cluster densities observed on Cu(100). These apparent discrepancies suggest that the large Cu clusters imaged with STM are unlikely to represent the catalytically active sites.

The exceptionally high reversibility of CO oxidation current densities observed over multiple current transients indicates a dynamic and reversible (re)­generation of active sites. To correlate this electrochemical reversibility with the underlying surface restructuring, we performed additional potential-step experiments with EC-STM on both Cu(111) and Cu(100), with focus on their structural evolutions upon stepping the potential back into the double-layer region, where no activity toward CO oxidation is observed. Figure 4 shows representative images of the electrode surfaces during CO oxidation and immediately after stepping back to −0.2 *V*_RHE_. On Cu(111), the anodically formed adatom clusters rapidly disappear upon the potential step back to −0.2 *V*_RHE_, with the terraces and islands contracting back laterally. This quasi-reversible reductive step is accompanied by the formation of a few additional vacancy islands, which form due to limited surface diffusion. Cu(100) exhibits a markedly different behavior, where a significant fraction of the small adatom clusters formed during CO oxidation persists after stepping back. Additionally, vacancy island formation is observed, similar to that on Cu(111). A corresponding reference experiment in Ar-saturated NaOH (Figure S6) shows that the reconstructed surfaces behave similarly upon the potential step back into the double-layer region, suggesting that the observed irreversibility for Cu(100) is most likely intrinsic to its surface dynamics, rather than driven by CO.

**Figure 4:**
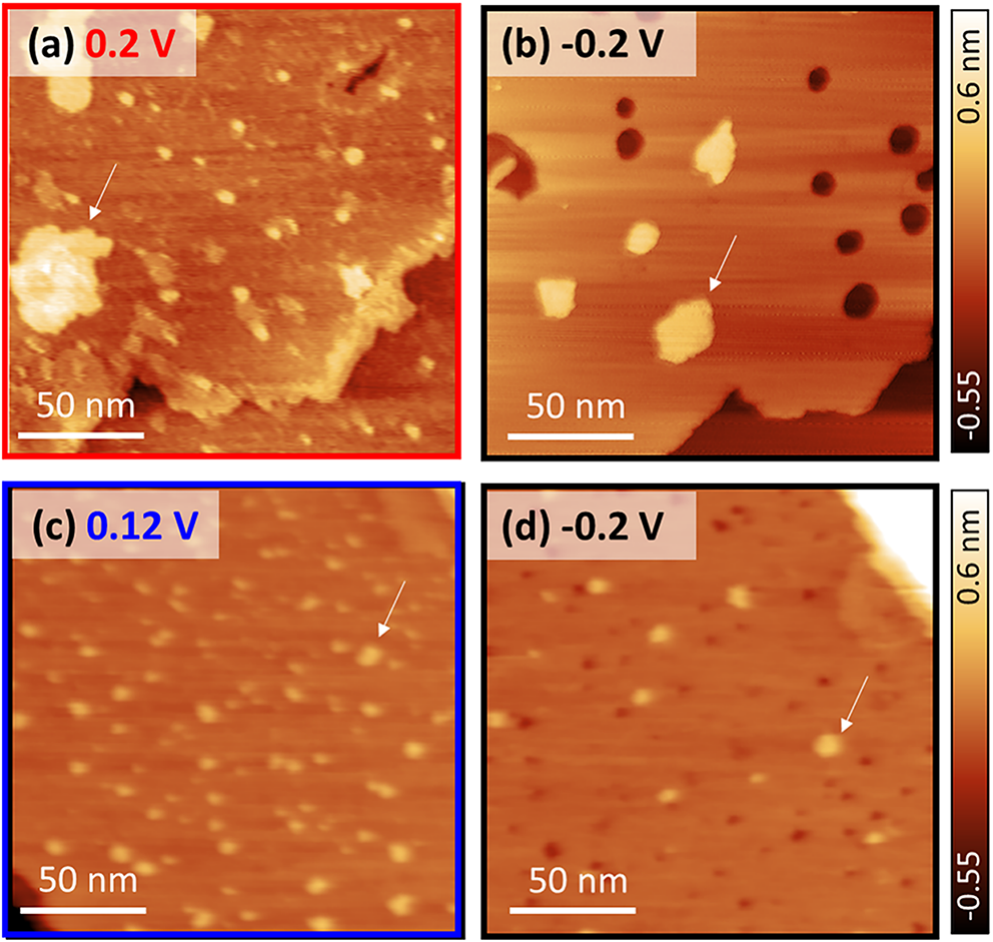
Reversibility of surface restructuring on Cu(111) and Cu(100) during CO electro-oxidation. EC-STM images of Cu(111) in CO-saturated 0.1 M NaOH at 0.2 *V*_RHE_ (a) and immediately after a potential step back to −0.2 *V*_RHE_, where no reaction occurs (b). Corresponding images of Cu(100) are shown at 0.12 V (c) and at −0.2 *V*_RHE_ (d). White arrows indicate the same spot on the surface during the reaction (a, c) and after the potential step back (b, d). *I*_tip_ = 1.0 nA (a, b), 0.8 nA (c, d), and *E*_tip_ = 0.15 V.

The persistence of nanoclusters on Cu(100), despite the absence of a corresponding irreversibility in the current transients, further supports our hypothesis that these clusters act as inactive spectator species rather than as catalytically active sites. Complementary CV experiments under alternating Ar and CO purging (Figure S7) confirm the (ir)­reversibility of the facet-dependent adsorption and restructuring characteristics.

To gain deeper insight into the extent of the irreversibility of the surface reconstruction on Cu(100), we conducted repeated potential-step EC-STM experiments into and out of the CO oxidation regime (Figure 5). Each potential step into CO electro-oxidation (Figure 5b,d,f) continuously induces nanocluster formation, while stepping back to −0.2 *V*_RHE_ leads to the appearance of small vacancy islands (Figure 5c,e). The cluster density gradually decreases with successive potential steps. Notably, the vacancy islands consistently disappear upon stepping into the CO oxidation regime. This observation suggests that similar to Cu(111), the topmost surface layer of Cu(100) undergoes lattice expansion under anodic conditions, resulting in the ejection of Cu adatoms and initial cluster formation. In contrast to Cu(111), we do not observe a reversible reformation of nanoclusters on Cu(100) after multiple potential steps. Nevertheless, given that the contraction of the reconstructed Cu layer typically results in partial reincorporation into the terrace and/or the attachment of previously ejected Cu adatoms at step edges, the systematic reappearance of the vacancy islands after each reductive potential step serves as indirect but conclusive evidence of the continuous generation of Cu adatoms under reaction conditions. Due to the limited time and spatial resolution of EC-STM, the direct observation of individual Cu adatoms remains inaccessible.

**Figure 5:**
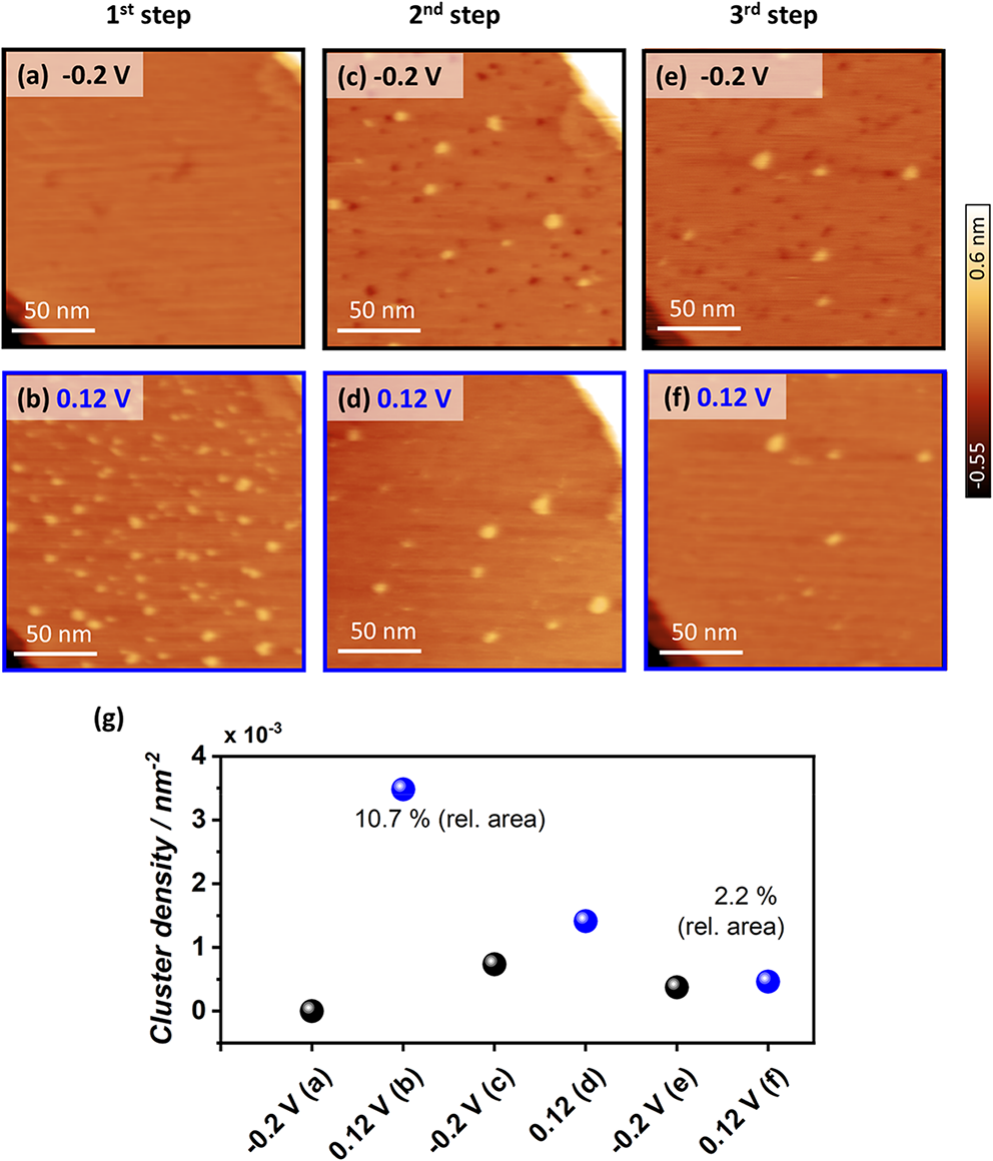
EC-STM images of repeated potential steps into the CO electro-oxidation regime on Cu(100). EC-STM images of (a, b) the first, (c, d) the second, and (e, f) the third potential step from −0.2 to 0.12 *V*_RHE_. All image sizes are 180 × 180 nm^2^. *I*_tip_ = 0.8 nA; *E*_tip_ = 0.15 V. (g) Quantitative cluster analysis of the EC-STM images.

Previous DFT calculations demonstrated that single Cu adatoms uniquely break the CO/OH^–^ scaling relation through selective weakening of OH^–^ binding while strengthening CO adsorption.^12^ This decorrelation effect is amplified compared to terrace- or cluster-sites due to the higher-energy electronic band of isolated adatoms. Analogous studies on Au surfaces show that CO preferentially stabilizes mobile adatom–CO complexes and enables their continuous regeneration.^37–39^ These effects are consistent with prior experimental and theoretical studies of CO adsorption on Cu,^15,17,18,23^ and, more generally, on transition metals.^40,41^ Altogether, these findings strongly indicate that single Cu adatoms are the primary catalytically active species under the reaction conditions. This assignment is inferred from a combination of EC-STM observations and DFT predictions, as direct imaging of the highly mobile adatoms is beyond the current spatial and temporal resolution abilities. However, larger clusters observed via EC-STM, as well as the resulting step-edge site density, can be reasonably excluded as the dominant active motif. While we cannot definitively exclude minor contributions from small oligomeric species (dimers and trimers), the similarity in their expected energetics and dynamics to those of monomeric adatoms suggests they would function within the same mechanistic framework.

Our EC-STM experiments reveal distinct differences in the effective population and stabilization of the isolated Cu adatoms under reaction conditions, which are largely governed by facet-specific adsorption behavior and the surface mobility/dynamics, as summarized in Figure 6. On Cu(111), OH^–^ adsorption in the presence of CO still occurs predominantly on terrace sites, which is evidenced by the formation of distinct dark patches in the EC-STM images, whereas the formed Cu adatoms are most likely stabilized by preferential CO adsorption. The spatial separation of the CO and OH^–^ adsorption sites suggests a bifunctional reaction mechanism. Therefore, the effective density of active sites can be considered higher due to the different possible adsorption sites of the two reactants. This mechanism is reflected in the consistently higher steady-state CO oxidation current densities observed for Cu(111). In contrast, on Cu(100), a similar reconstruction-induced generation of Cu adatoms occurs but without the formation of distinct OH^–^ patches on the terrace. Instead, CO and OH^–^ likely compete for coadsorption at the undercoordinated Cu adatom sites. This coadsorption scenario leads to an effectively lower active site density. Furthermore, Cu(100) exhibits a reduced capacity to reincorporate ejected adatoms back into the surface, which leads to the formation of irreversible nanoclusters that can additionally act as spectator species. Consequently, the density of active Cu adatom sites and the competitive interaction of reactants on Cu(100) correlate with its lower steady-state activity compared to Cu(111).

**Figure 6:**
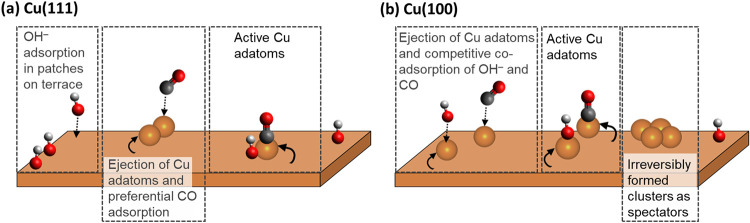
Schematic illustration of the proposed differences in the CO electro-oxidation mechanisms on Cu(111) and Cu(100). (a) On Cu(111), OH^–^ adsorption predominantly occurs on the terraces, which leads to surface reconstruction and ejection of Cu adatoms that are subsequently stabilized by preferential CO binding. (b) On Cu(100), a similar restructuring takes place; however, OH^–^ does not adsorb in distinct patches on the terraces, when CO is present. Instead, competitive coadsorption of CO and OH^–^ likely occurs at the formed undercoordinated Cu adatoms. In addition, nanoclusters are formed irreversibly, which presumably are inactive spectator species.

To evaluate whether the dynamically maintained population of isolated adatoms under reaction conditions could sustain the observed activity, we estimated the required coverages based on the measured steady-state current densities (Table S1). At turnover frequencies (TOFs) of 100–1000 s^–1^, the required adatom coverage (10^–3^ to 10^–4^ ML) remains well below typical nucleation thresholds (10^–2^ ML).^42–45^ While this required coverage corresponds to only a minor fraction of surface Cu atoms, it exceeds the equilibrium adatom concentration at room temperature (approximately 10^–9^ ML)^43^ by several orders of magnitude, indicating that the applied potential and ongoing reaction fundamentally alter the adatom population. The concurrent observation of a stable catalytic current (Figure 2) alongside continuous morphological evolution (Figure 3) demonstrates that the system maintains a kinetically controlled adatom population through a dynamic balance between restructuring-driven generation and CO-stabilized consumption.

Thus, isolated Cu adatoms can realistically sustain the observed catalytic activity without requiring cluster formation, consistent with the observed decrease in the cluster density on Cu(100). This reinforces the assignment of single Cu adatoms as the central active motif and offers a nuanced understanding of their role in driving the high catalytic activity. It also provides a consistent explanation for the observed facet-dependent activity trends and highlights the necessity of considering dynamic surface transformations when structure–activity relationships are established in electrocatalysis.

## Conclusion

Our combined electrochemical and EC-STM analysis demonstrates that CO electro-oxidation on both Cu(111) and Cu(100) involves the reaction-induced formation of undercoordinated Cu adatoms, which serve as the catalytically active species. Although both facets undergo nanometer-scale restructuring with the formation of small clusters that consist of multiple adatoms, the morphological evolution of these adatom clusters is distinctly facet-dependent. Cu(111) shows quasi-reversible restructuring, maintaining a high density of small nanoclusters across multiple potential steps. In contrast, Cu(100) forms nanoclusters that evolve less reversibly, with an accompanying gradual decrease in the overall cluster density over time and successive potential steps.

Importantly, our results reveal that catalytic activity is not directly correlated to the total nanocluster coverage or density. Rather, the effective population of catalytically active Cu adatoms is determined by the interplay between restructuring dynamics and facet-specific adsorption behavior of the reactants. EC-STM images of Cu(111) under reaction conditions support a bifunctional mechanism, where OH^–^ adsorbs on terrace sites and CO binds to adatoms, resulting in a high effective active site density. Conversely, on Cu(100), competitive coadsorption of CO and OH^–^ on the same undercoordinated sites limits accessibility, which leads to a lower effective active site population.

These findings establish a mechanistic connection among surface restructuring, local adsorption environments, and facet-dependent catalytic performance. They emphasize that static structure–activity models fail to capture the dynamic evolution of catalyst surfaces, highlighting the necessity for design strategies that explicitly consider morphological and structural changes under the reaction conditions.

## Methods

### Electrode Preparation

Cu­(111) and Cu(100) single-crystal electrodes (Mateck, Jülich) were first mechanically polished using diamond pastes with decreasing particle sizes (3 μm, 1 μm, and 0.25 μm; ESCIL), followed by electropolishing in 60% phosphoric acid (prepared from 85% EMSURE H_3_PO_4_, Merck) at 1.8 V versus a copper counter electrode. The electrodes were then thoroughly rinsed with Milli-Q water (>18 MΩ cm, Merck). Prior to STM measurements, the polished crystals were additionally annealed at 840 °C under a hydrogen atmosphere in a custom-built horizontal tube furnace. After annealing, the electrodes were transferred directly into an Ar-filled glovebox (MBraun MB 200 MOD) without exposure to ambient air.

### Electrochemical Measurements

Electrochemical experiments were carried out in a three-electrode configuration using a Teflon body to prevent contamination of glassware in the alkaline environment. The electrolyte (0.1 M NaOH, Merck, 99.99% trace metal basis) was either purged with Ar (99.990%, Messer, Austria) to eliminate dissolved oxygen or saturated with CO (99.995%, Linde, Germany) via an all-copper gas line to avoid contamination. A carbon rod served as the counter electrode, and potentials were recorded against either a PTFE-bound activated carbon quasi-reference electrode (carbon black, 99%, Cabot, U.S.), which is described in detail in ref 46 or a commercial reversible hydrogen electrode (RHE, Hydroflex, Gaskatel). All voltammetric and transient experiments were conducted in a hanging meniscus configuration by using a Biologic VSP 300 potentiostat, and the measured potentials are reported on the RHE scale.

### *In Situ* EC-STM

Electrochemical STM measurements were performed with a Keysight 5500 instrument in an Ar-filled glovebox (MB 200 MOD, MBraun, Germany). The single-crystal electrodes were mounted in a custom-designed EC-STM cell made of Kel-F, equipped with PTFE-bound activated carbon as both quasi-reference and counter electrodes. Prior to imaging, both Cu(111) and Cu(100) were immersed in Ar-saturated 0.1 M NaOH electrolyte, with Cu(111) held at an open-circuit potential and subsequently reduced to remove thin surface oxides, while Cu(100) was immersed under potential control at −0.2 *V*_RHE_. For CO electro-oxidation experiments, the Ar-saturated electrolyte was then exchanged with a CO-saturated solution while maintaining the applied potential. The glovebox was operated under slight overpressure, which suppressed electrolyte evaporation and stabilized the dissolved CO concentration. CO saturation was carried out inside the glovebox using an all-copper gas line, ensuring that a CO-rich atmosphere was also established above the EC-STM cell. To prevent depletion of dissolved CO under electro-oxidation conditions, the duration of each measurement series was limited to a maximum of 30 min due to the restricted CO solubility and electrolyte volume of the cell. Imaging parameters (tunneling current and bias voltage) are reported in the corresponding figure captions. STM data were processed and analyzed using Gwyddion software.^47^

## Supplementary Material



## Data Availability

The data that support the findings of this study are openly available in InvenioRDM at 10.48323/q7mt9-ftz36.

## Notes

Details on the steady-state current densities of the transient measurements; line profiles and further EC-STM images on the CO oxidation and OH adsorption of Cu(111) and Cu(100); cyclic voltammetry experiments under alternating Ar and CO purging conditions; quantitative validation of the observed catalytic activity by estimating the required adatom coverages from the measured steady-state current densities (PDF)
