# Supplementary material for: Facet-Dependent Restructuring and Catalytic Activity of Cu Single Crystals during CO Electro-Oxidation
Source: ACS Nano. 2025 Dec 9;19(50):42310–8. doi: 10.1021/acsnano.5c13881 (PMC12752700; doi:10.1021/acsnano.5c13881)
Supplement: Supplementary file 1 [file nn5c13881_si_001.pdf]

Supporting Information

Facet-Dependent Restructuring and Catalytic Activity of Cu Single Crystals during CO Electro-Oxidation

Matthias Leitner, Francesc Valls Mascaró, Andrea Auer, and Julia Kunze-Liebhäuser*

Institute of Physical Chemistry, University Innsbruck, Innrain 52c, Innsbruck, 6020, Austria

E-mail: andrea.auer@uibk.ac.at

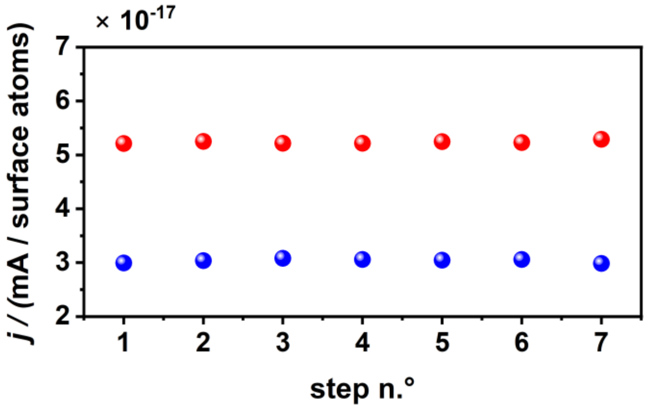

Figure S1: Steady-state current density normalized to the number of surface atoms for Cu(111) (red) and Cu(100) (blue).

To account for the difference in surface atom density between Cu(111) and Cu(100), the steady-state current densities of each potential step were normalized to the nominal number of surface atoms for the (1 x 1) non-reconstructed (111) and (100) crystal facets. These values are derived from the specific atomic arrangement of the two surface orientations, yielding surface atom densities of 1.78×10^{15} atoms cm^{-2} for Cu(111) and 1.54×10^{15} atoms cm^{-2} for Cu(100).

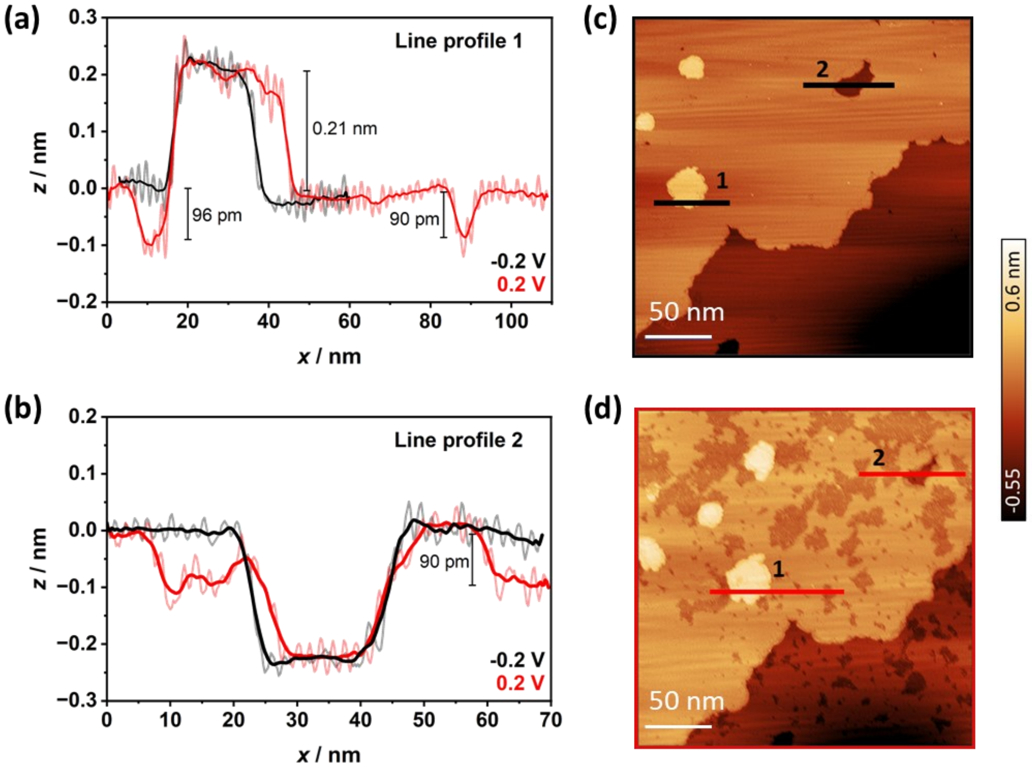

Figure S2: (a, b) Line profiles of Cu(111) in CO-saturated 0.1 M NaOH, comparing the height and width of the same adatom (1) and vacancy island (2) at −0.2 V_{RHE} (black line) and 0.2 V_{RHE} (red line), respectively. The dark patches that appear during CO oxidation at 0.2 V exhibit a characteristic height of 90 to 100 pm. (c, d) Corresponding EC-STM images (250 nm x 250 nm; I_{tip} = 1 nA, E_{tip} = 0.15 V) with the numbered white lines indicating the locations of the line profiles shown in panels (a) and (b).

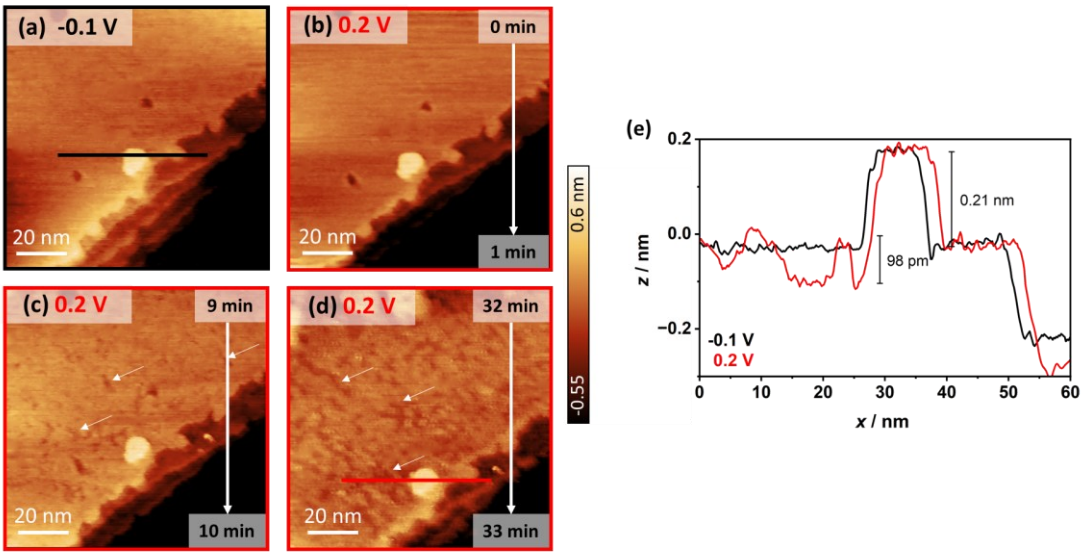

Figure S3: EC-STM imaging of Cu(111) during OH^- adsorption in CO-free 0.1 M NaOH. Cu(111) at (a) −0.1 V_{RHE} and (b-d) during a potential step into the OH^- adsorption regime at 0.2 V_{RHE}. White arrows mark dark patches associated with OH^- adsorption. All image sizes are 100 × 100 nm^2. I_{tip} = 1.4 nA, E_{tip} = 0.15 V. (e) Corresponding line profiles of Cu(111) at − 0.1 V_{RHE} (black line) and 0.2 V_{RHE} (red line), marked by the horizontal lines in (a) and (d), respectively.

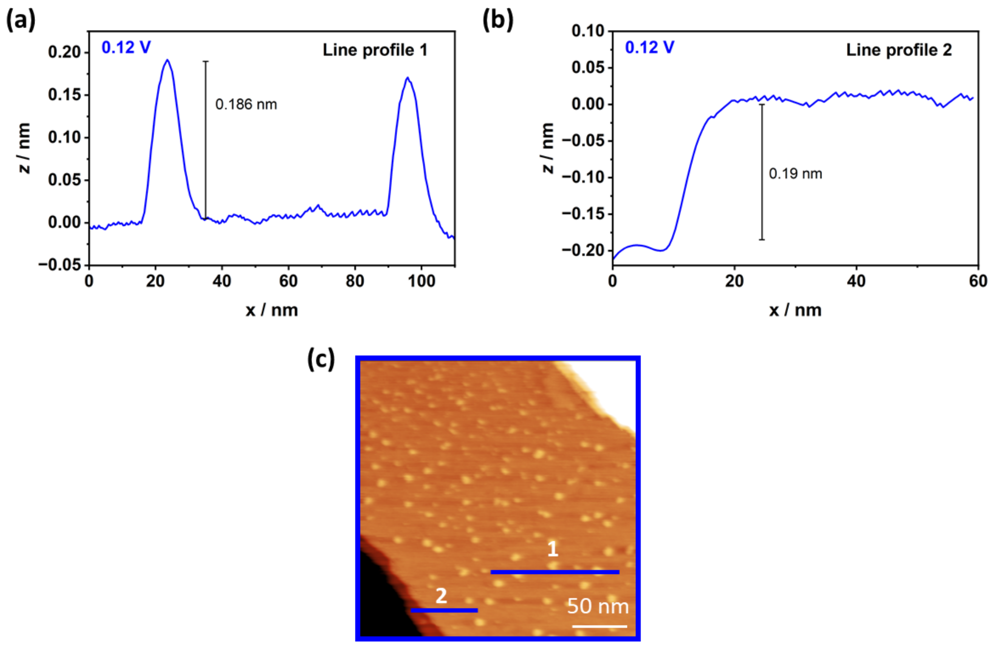

Figure S4: (a,b) Line profiles of Cu(100) in CO-saturated 0.1 M NaOH at 0.12 V_{RHE} (blue lines). The heights of two Cu adatom clusters (1) are compared to the monoatomic step height (2). (c) Corresponding EC-STM image (250 nm x 250 nm; I_{tip} = 0.8 nA, E_{tip} = 0.15 V) with the numbered blue lines indicating the locations of the line profiles shown in panels (a) and (b).

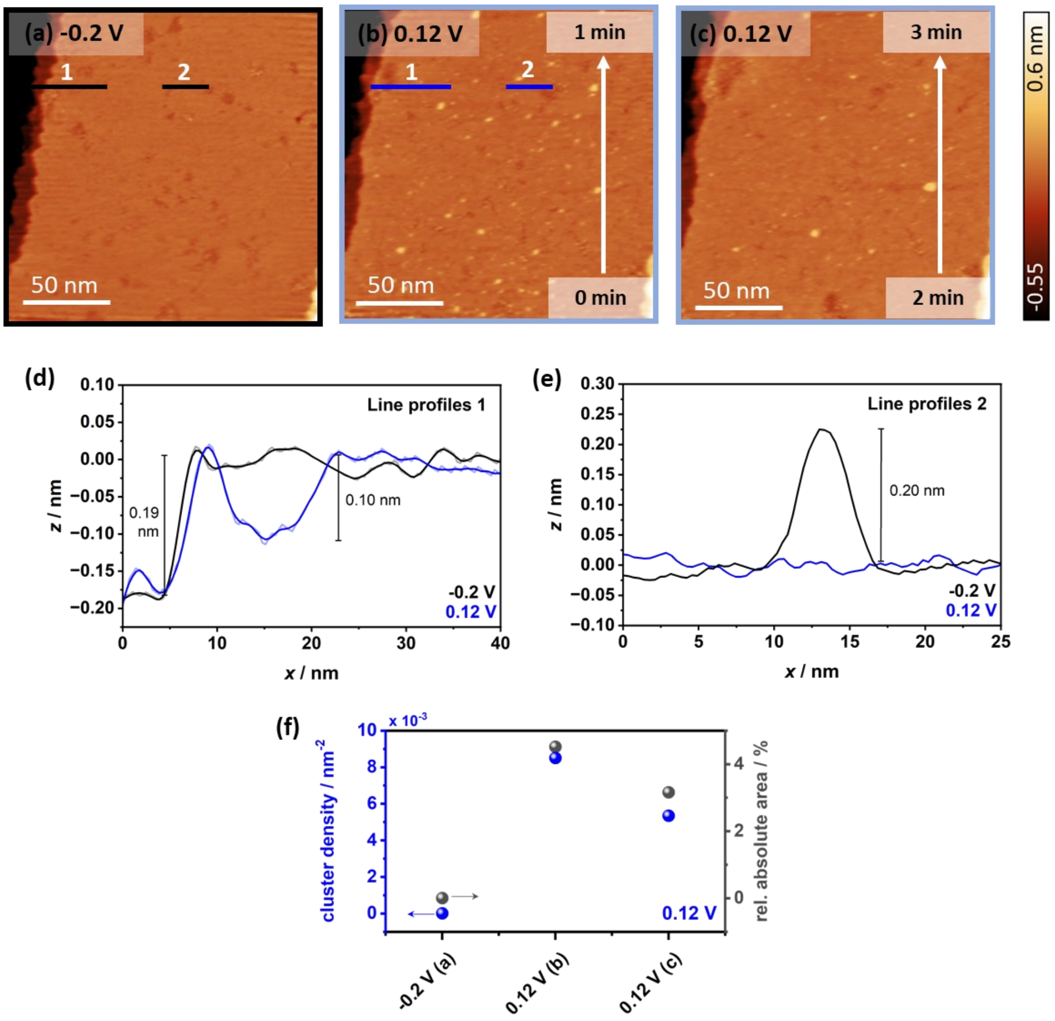

Figure S5: EC-STM images of Cu(100) during OH^- adsorption in CO-free 0.1 M NaOH at (a) −0.2 V_{RHE} and (b-c) during a potential step into the OH^- adsorption regime at 0.12 V_{RHE}. Line profiles compare the height of a monoatomic step (d) and a Cu adatom cluster (e) at −0.2 V_{RHE} and 0.12 V_{RHE}. The locations of the line profiles are shown in the EC-STM images (a,b). All image sizes are 180 × 180 nm^2. I_{tip} = 1.26 nA , E_{tip} = 0.15 V. (f) Quantitative cluster analysis of the shown EC-STM images, displaying cluster density (blue, left y-axis) and relative cluster area (grey, right y-axis). Colored arrows indicate the corresponding y-axis.

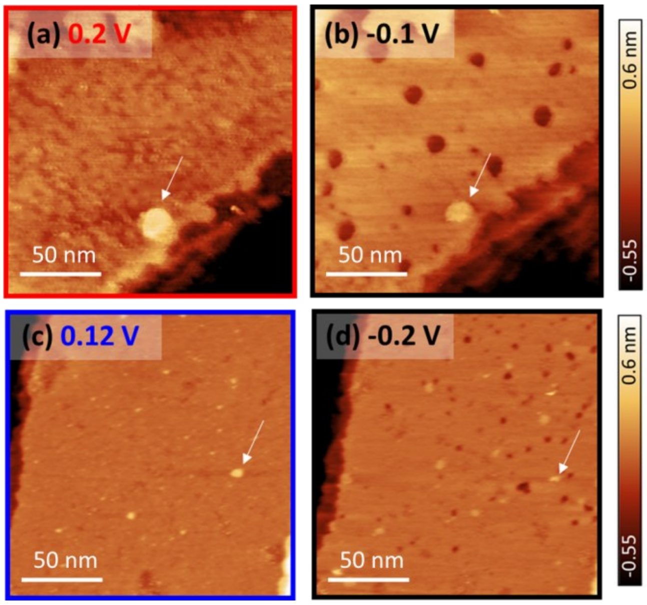

Figure S6: Reversibility of the OH-induced reconstruction of Cu(111) and Cu(100) in Ar-saturated 0.1 M NaOH. EC-STM images of Cu(111) in the OH adsorption regime at 0.2 V_{RHE} (a) and immediately after a potential step to −0.1 V_{RHE}, where no reaction occurs (b). Corresponding images of Cu(100) at 0.12 V (c) and at −0.2 V_{RHE} (d). White arrows indicate the same spot in the OH adsorption regime (a,c) and after the potential step back into the double layer region (b,d). Images sizes are 100x100 nm^2 (a,b) and 180x180 nm^2 (c,d); I_{tun} = 1.4 nA (a,b), 1 nA (c,d); E_{tip} = 0.15 V_{RHE}.

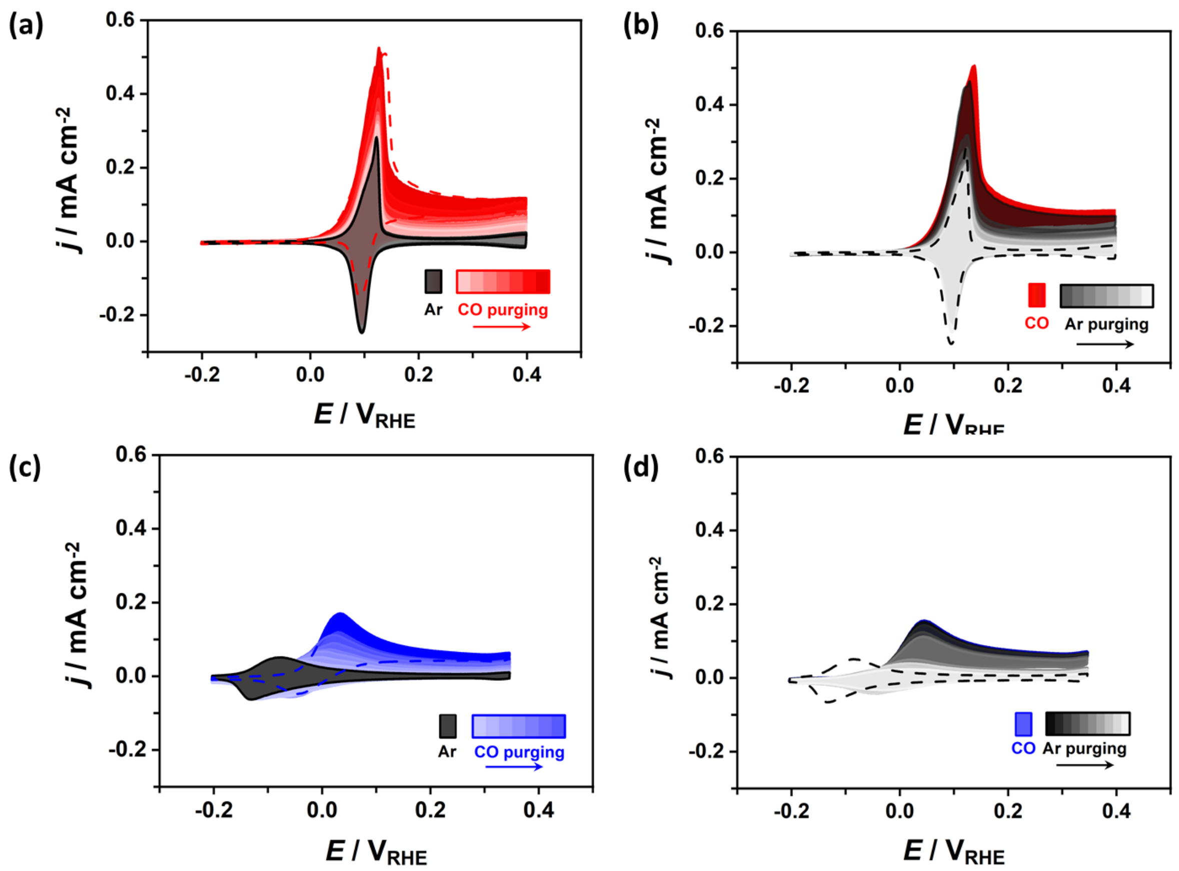

Figure S7. Cyclic voltammograms (CVs) of Cu(111) (a,b) and Cu(100) (c,d) recorded in 0.1 M NaOH under alternating Ar and CO purging. Sequential CVs, indicated by the color scale, show the evolution of the OH^- adsorption region and CO oxidation currents. The dotted red (Cu(111)) and blue (Cu(100)) traces in (a) and (c), respectively, correspond to the voltammograms at the highest CO concentration. The dotted black traces in (b) and (d) indicate the OH^- adsorption features prior to any CO exposure. Scan rate: 100 mV s^{-1}.

To complement the EC-STM observations in Fig. 4, we recorded CVs under alternating Ar and CO purging (Fig. S7) to directly probe how CO adsorption affects the characteristic OH^- adsorption features of the two Cu facets in 0.1 M NaOH.

For Cu(111) (Fig. S7a-b), the sequential voltammetric response (dark red corresponds to the highest CO concentration) shows that the OH^- adsorption feature remains visible and at the same potential while CO oxidation currents evolve. It is fully reversible upon switching between Ar- and CO-saturated electrolyte, with the OH^- adsorption peak around 0.1 V_{RHE} fully recovering once CO is removed. This indicates that OH^- adsorption energetics are unaffected by CO and confirms the high reversibility of the process. In contrast, Cu(100) (Fig. S7c-d) exhibits a pronounced potential shift of the OH^- adsorption peak upon CO introduction (dark

blue corresponds to the highest CO concentration). Even after returning to Ar purging, the original voltammetric features are not completely restored, suggesting that CO adsorption alters the local surface structure or site distribution. This partial irreversibility in the voltammetric response correlates with the EC-STM observations (Fig. 4), which revealed a corresponding loss of reversibility in the structural reorganization of Cu(100) after CO electro-oxidation.

Table S1. Quantitative estimation of isolated adatom concentrations and corresponding turnover frequency (TOF) required to account for the measured steady-state current densities for CO oxidation on Cu(111) and Cu(100).

Cu111

current density / mA cm^{-2}	0.1
current density / A cm^{-2}	0.0001
surface atom density ((1x1), 1 ML)	1.780E+15
rate CO_2 production / mol/cm^{-2}/s	5.182E-10
rate CO_2 production / molecules/cm^{-2}/s	3.223E+14

**TOF /k s^{-1}**	**Required adatom concentration**	**ML**	
1	3.223E+14	0.1811	above cricital nucleation density
10	3.223E+13	0.0181	close to cricital nucleation density
100	3.223E+12	0.0018	under cricital nucleation density
1000	3.223E+11	0.0002	under cricital nucleation density

Cu100

current density / mA cm^{-2}	0.05
current density / A cm^{-2}	0.00005
surface atom density ((1x1), 1 ML)	1.540E+15
rate CO_2 production / mol/cm^{-2}/s	2.591E-10
rate CO_2 production / molecules/cm^{-2}/s	1.612E+14

**TOF /k s^{-1}**	**Required adatom concentration**	**ML**	
1	1.612E+14	0.1047	above cricital nucleation density
10	1.612E+13	0.0105	close to cricital nucleation density
100	1.612E+12	0.0010	under cricital nucleation density
1000	1.612E+11	0.0001	under cricital nucleation density
